# Case report: Condylar metastasis from hepatocellular carcinoma: An uncommon case report and literature review

**DOI:** 10.3389/fonc.2022.1085543

**Published:** 2023-01-11

**Authors:** Xiaojie Liu, Yanshan Liu, Jian Sun, Ningyi Li, Cunhui Fan, Liqiang Chen

**Affiliations:** ^1^ Department of Stomatology, The Affiliated Hospital of Qingdao University, Qingdao, Shandong, China; ^2^ School of Stomatology of Qingdao University, Qingdao, Shandong, China

**Keywords:** hepatocellular carcinoma, condylar metastasis, condyle, case report, literature review

## Abstract

**Background:**

Patients with hepatocellular carcinoma are often affected by metastases, but condylar metastasis is particularly rare.

**Case presentation:**

A 51-year-old man with a history of hepatocellular carcinoma requested treatment for facial pain. Computed tomography indicated that the condylar bone has been destroyed and fractured. Pathology confirmed condylar metastasis from hepatocellular carcinoma. Complete metastasectomy and condylar reconstruction were performed to preserve his facial appearance. No local recurrence or distant metastasis was found at 8 months of follow-up.

**Conclusion:**

The condyle can be a metastatic site of hepatocellular carcinoma, regardless of its rarity. Long-term comprehensive surveillance and follow-up are needed for patients with hepatocellular carcinoma. The presence of solitary mass does exclude the possibility of metastatic cancer for these patients, and postoperative imaging and pathological diagnosis are important to determine its origin. If patients’ physical condition permits, the mass can be completely excised, and the physiological function can be restored and reconstructed.

## 1 Introduction

Hepatocellular carcinoma (HCC) is a common malignant tumor and has the third highest mortality rate in global malignant tumor-related diseases (1). The most common metastatic site of HCC is the lung, and metastasis from HCC to the condyle is extremely rare. About 1% of oral malignant tumors can be attributed to metastatic cancer, and most of them are secondary to lung, breast, prostate, and other types of cancer. We report a case of condylar metastasis from HCC and reviewed relevant literature, aiming to collect clinical characteristics and provide a reference for the treatment of these patients.

## 2 Case report

A 51-year-old male patient was hospitalized in our hospital complaining of “right facial pain for 6 months”. The patient felt sharp radiating pain in the right preauricular area 6 months before, and self-administered painkillers were effective. The pain worsened gradually, so he came to the Affiliated Hospital of Qingdao University for further treatment. The patient had a history of HCC 9 years ago and underwent surgery. He received re-operation for a recurrence of HCC 5 years 9 months ago. Other history included hepatitis B virus, hypertension, and diabetes, all of which were currently controlled by oral medication. The patient and his family had no history of the tumor.

**Figure 1:**
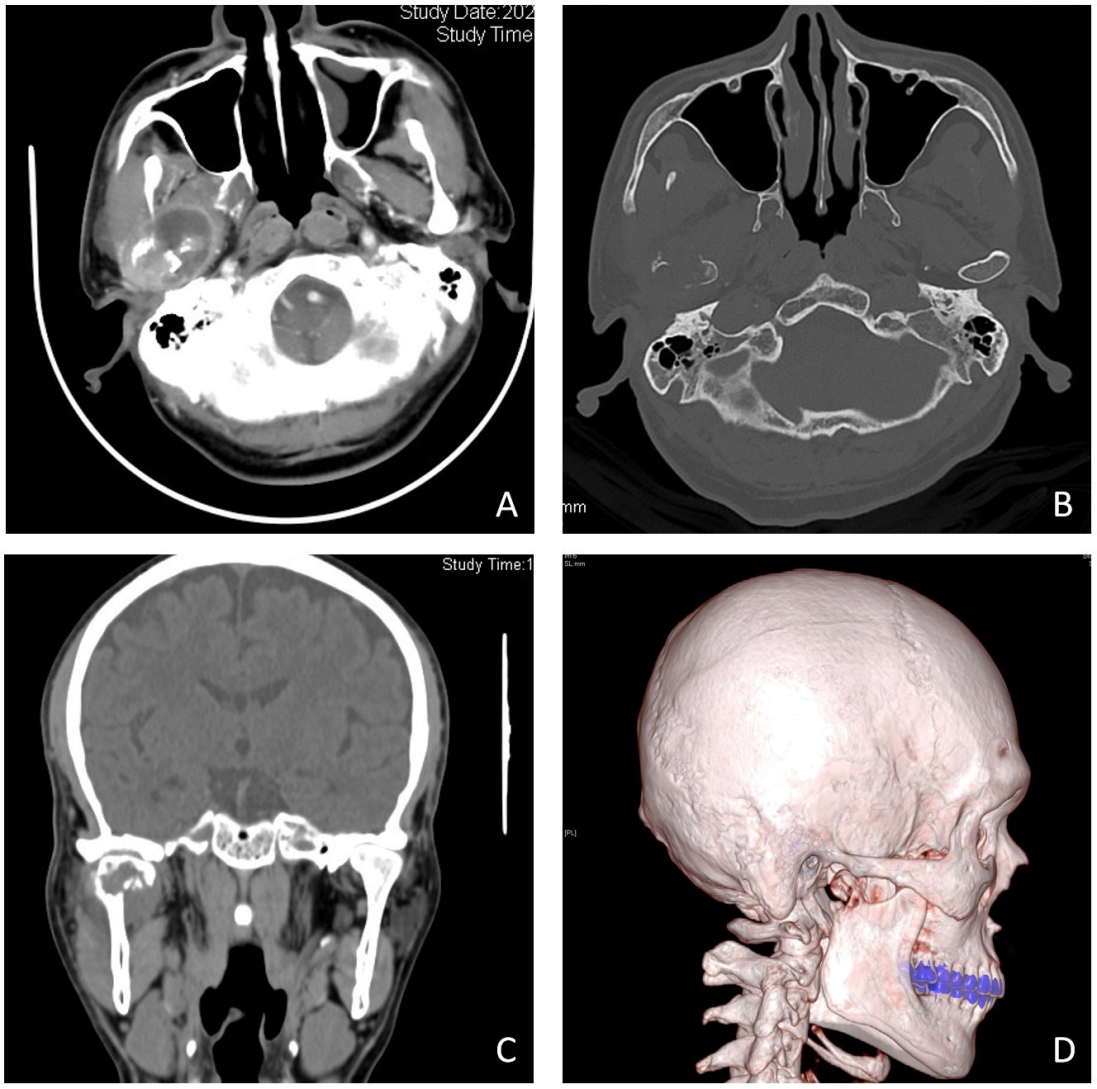
Imaging manifestations of condylar metastasis in contrast-enhanced (CT) and three-dimensional imaging. Contrast-enhanced CT shows a mass **(A)**, bone-window imaging indicates that the condylar bone has been destroyed **(B)**, and coronal position imaging and three-dimensional reconstruction imaging indicate that the condylar bone has been destroyed and fractured **(C, D)**.

Clinical examination revealed bilateral symmetry of the oral and maxillofacial region, with obvious tenderness in the right temporomandibular joint (TMJ) region, and no obvious abnormalities in mouth opening and occlusal relationship were found. Computed tomography (CT) showed occupancy in the right TMJ subregion, destruction of the condylar bone, and pathological fracture, demonstrating a high possibility of neoplastic lesions (**Figure 1**). Contrast-enhanced CT of the upper and lower abdomen and chest showed no obvious abnormalities. Laboratory examinations showed high alkaline phosphatase and adenosine deaminase. Our initial suspicion is condylar metastasis from HCC.

**Figure 2:**
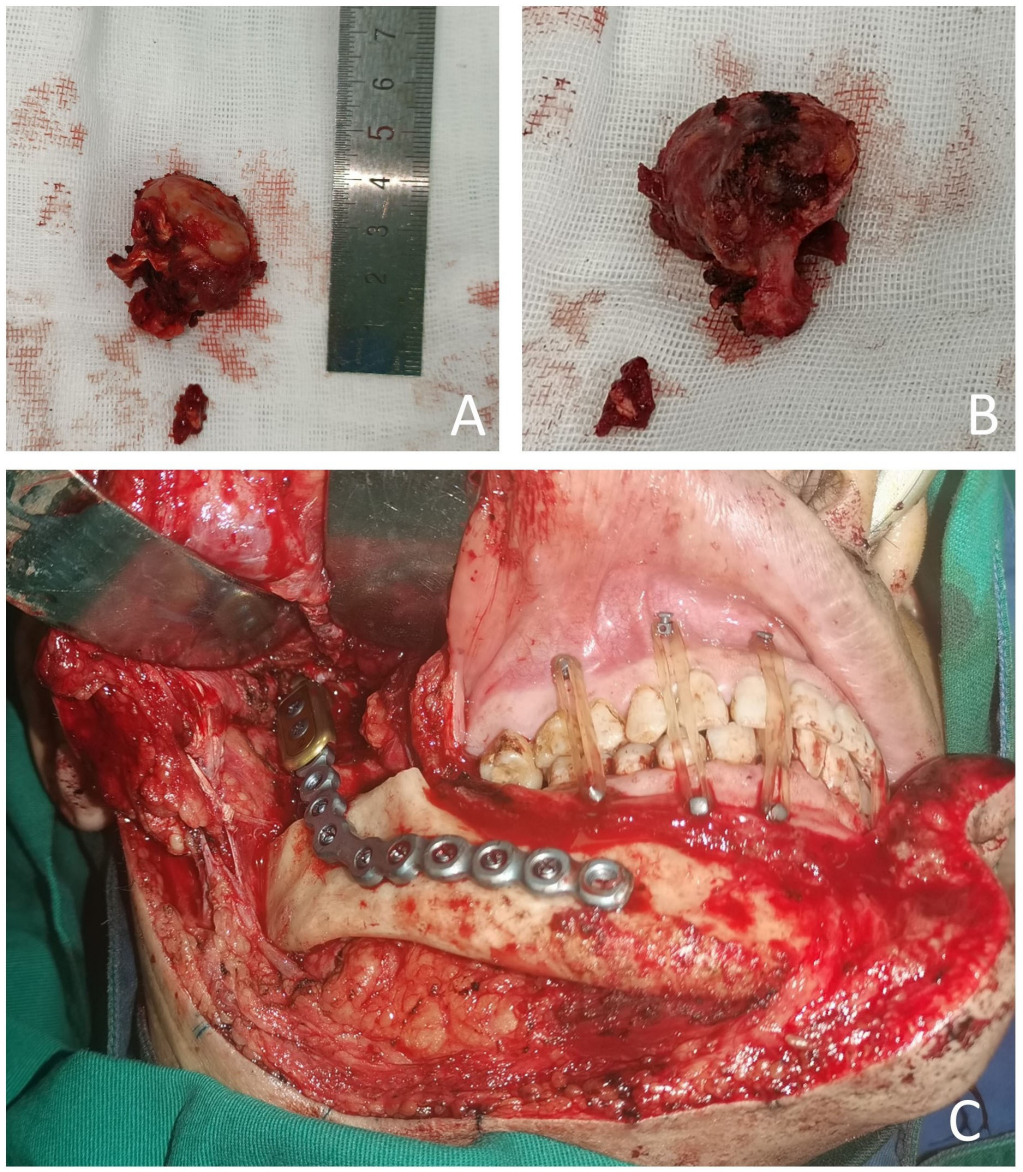
Imaging of surgical procedure **(A–C)**.

**Figure 3:**
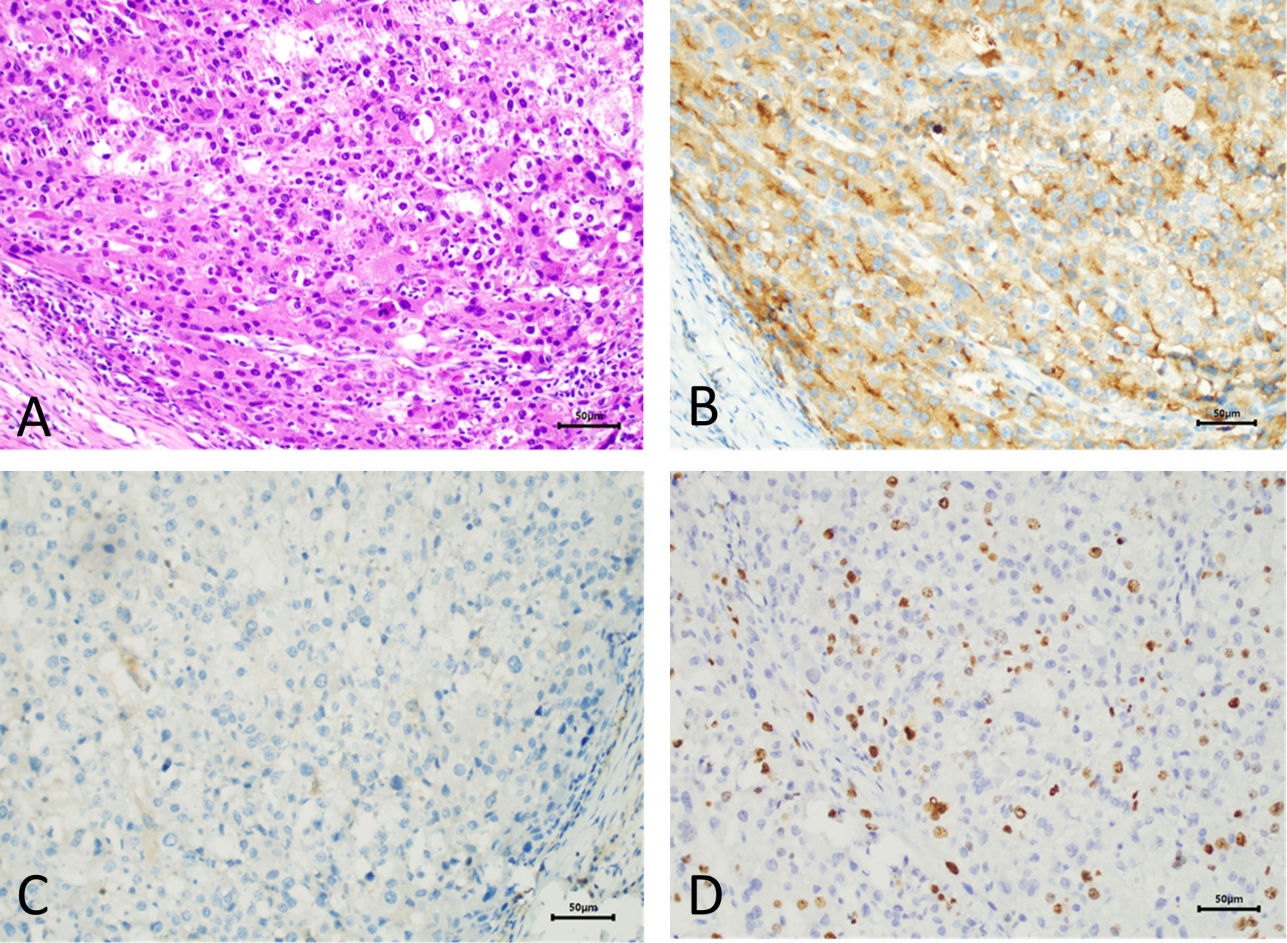
Pathological manifestations of hepatocellular metastasis. Hematoxylin–eosin staining (×200) **(A)**. Immunohistochemistry: GPC-3 (×200) **(B)**. Immunohistochemistry: hepatocyte (×200) **(C)**. Immunohistochemistry: Ki-67 (×200) **(D)**.

The patient requested the removal of the mass. Complete metastasectomy and condylar reconstruction were performed while preserving the facial appearance. The mass was found in the condylar and measured approximately 40 mm × 30 mm × 18 mm. The mass is gray and red in the section, and the surface of the mass was rough (**Figure 2**). Postoperative pathology showed that the right condyle mass was a poorly differentiated carcinoma with necrosis, which was diagnosed as hepatocellular carcinoma metastasis with no involvement of the resection margin. Immunohistochemistry revealed positive reactivity to CKpan, GPC-3, Ki-67 (20%), PD-L1 (22C3) (CPS ≈ 5), and negative reactivity to hepatocyte, arginase-1, alpha-fetoprotein (AFP), CDX-2, CD31, and CK7 (**Figure 3**). The immunohistochemical primary antibodies are available in a ready-to-use 6-ml reagent. The patient recovered well after surgery and CT review. PET-CT showed a patchy hypodense shadow under the diaphragm in the posterior hepatic apex, mild metabolism, initial SUVmax about 3:1, and delayed SUVmax about 2.4. The patient was transferred to the oncology department after surgery for radiotherapy to the right mandibular region, DT45GY, for a total of 15 sessions. The patient is currently in his eighth month postoperatively with a good recovery and is still being followed up.

## 3 Discussion

Metastases to the jaw account for less than 1% of all oral malignancies (2). Metastatic HCC to the jaws is very rare, with a total of only 41 cases of metastatic hepatocellular carcinoma (MHCC) reported in English literature (3). Related studies have shown that most metastatic cancers of the jaw metastasize to the mandible (82%), with the molar region being the most frequent site, followed by the premolar region and the angle–ramus (4, 5). Condylar metastasis accounts for only 3.5% of maxillary metastases. The low incidence may be related to the small amount of local bone marrow in the condyle, which has an independent vascular supply from the circulating penetrating branches of the maxillary and superficial temporal artery. Further, the presence of a limiting osseous plate that isolates the condylar marrow cavity from the spongiosa of the mandible properly prevents the spread of metastatic cells (6). Because reports of metastatic carcinoma of the condyle are few, we summarized 10 such cases searched in PubMed and analyzed their origin, clinical features, and prognosis (see **Table 1**). The keywords were “condylar/condyle” and “metastasis/metastases” restricted to English articles with a cutoff date of 10 October 2022. Analysis of the relevant literature reveals that cases of hepatocellular carcinoma metastasis to the condyle are rare. The mechanism of maxillofacial region metastasis from HCC is not yet fully understood, but current research suggests that there are two pathways from the liver to the maxillofacial region (12), which initially reach the lungs *via* the hepatic artery and portal vein and finally reach the maxillofacial region. Vitale et al. showed that HCC can spread to Baston’s plexus (the plexus connects the azygos and hemiazygos veins with the vertebral venous plexus) and then bypass the filtration of the lungs to reach the maxillofacial region.

**Table 1:** Clinical characteristics of cases with condylar metastasis from articles.

Ref	Age (year) Gender	From	Initial examination	Further examination	Number/side	Combined metastasis	Treatment	Outcome (month)
Shinpei Matsuda et al. (7)	83/F	Lung	Symptoms	CT/MRI	1/R	Spine, brain, and cervical lymph node​	Chemotherapy	No progression
Ya-ting Q et al. (8)	49/M	Bladder	Symptoms	CT/PET-CT	1/L	Retroperitoneal lymph nodes/clavicle	Metastasectomy/chemotherapy	Died 6 months later
Ya-ting Q et al.	85/M	Prostate	Symptoms	CT/PET-CT	1/R	Retroperitoneal lymph nodes/clavicle	Metastasectomy/chemotherapy	Died 12 months later
Ya-ting Q et al.	62/F	Lung	Symptoms	CT/PET-CT	1/L	Rib/ilium	Chemotherapy	Died 6 months later
Ya-ting Q et al.	53/M	Penis	Symptoms	CT/PET-CT	1/R	Several metastasis	Chemotherapy	Died 3 months later
Ya-ting Q et al.	64/M	Colon	Symptoms	CT/PET-CT	1/L	Several metastasis	Chemotherapy	Died 3 months later
Ya-ting Q et al.	47/F	Breast	Symptoms	CT	1/R	–	Metastasectomy/radiotherapy	Alive
Scolozzi P et al. (9)	72/M	Lung	Symptoms	CT/MRI	1/R	–	radiotherapy	Died 6 months later
Freudlsperger C et al. (10)	75/M	prostatic	Symptoms	CT/MRI	1/L	–	Chemotherapy/radiotherapy	–
M. G. Kaufmann et al. (11)	48/F	lung	Physical examinations	CT/PET-CT	1/L	–	Metastasectomy/radiotherapy	20 months alive
Present case	51/M	liver	Symptoms	CT/PET-CT	1/R	–	Metastasectomy/radiotherapy	No progression

Radiographically, metastatic cancers usually present as destructive irregular “moth-eaten” radiolucency. Some tumors can induce reactive new bone formation, producing a mixture of radiopaque and radiolucent lesions, which may be mistaken for a fibro-osseous lesion. This pattern is characteristically seen with metastatic breast and prostate cancer (10). In this case, CT indicated that the right condylar bone had been destroyed and fractured, and the condyle tumor occurred after multiple recurrences of hepatocellular carcinoma, so metastatic carcinoma was highly suspected. Although the condyle tumor, in this case, is secondary to HCC, the condyle tumor may also be the first manifestation of HCC. Teshigawara et al. (13) have reported that oral masses were found in 59% of patients prior to the diagnosis of hepatocellular carcinoma. Therefore, patients with an isolated condylar tumor should be concerned about their past medical history and laboratory tests. A history of hepatitis B with a significantly elevated AFP is highly suggestive of primary HCC. However, 30% of HCC patients remain negative for AFP. The final diagnosis of HCC metastasis still relies on postoperative histopathological examination and immunohistochemistry. Immunohistochemical staining is crucial to determine the nature of the mass and to identify primary and secondary tumors. Previous cases diagnose HCC metastases using hepatocyte differentiation markers with different sensitivities and specificities, such as arginase-1, hepatocyte paraffin 1 (Hep-1), polyclonal carcinoembryonic antigen (CEA), and GPC-3. Among these immunostains, arginase-1 (Arg-1) is the most sensitive marker, showing high sensitivity even in hypofractionated HCC and scirrhous HCC (14), and decreased or absent Arg-1 expression may promote HCC metastasis. GPC-3 is highly expressed in HCC but not in normal tissues and therefore can be used as a specific detection marker for HCC. The combined application of Arg-1, GPC-3, and Hep-1 is crucial in both the diagnosis and differential diagnosis of HCC, and the high specificity of GPC can effectively compensate for the lack of staining of Arg-1 and Hep-1. In this case, the postoperative pathological diagnosis confirmed metastatic hepatocellular carcinoma. The typical histological pattern and positive immunohistochemistry for CK and GPC-3 supported this diagnosis. Although hepatocyte and arginase-1, the immunohistochemical markers associated with hepatocellular carcinoma, were negative for AFP, the diagnosis was still confirmed by the typical morphology and clear clinical history. Ki-67 indicated a low proliferation index and low expression of PD-L1 (22C3), both of which can provide certain references for clinicians in systemic therapy and immunotherapy.

One study showed that the average survival time for patients after HCC diagnosis was 12.4 compared to 6.1 months after jaw metastasis. Oral metastases of hepatocellular carcinoma are often part of the HCC metastases, and when oral metastasis from HCC is found, it often means that the patient is in an advanced stage of HCC (15). The risk of local recurrence in the short term is high and the prognosis is poor for mandibular metastases, so palliative care is often required for metastatic HCC. Currently, targeted molecular therapies such as sorafenib occupy a central position in the systemic treatment of advanced hepatocellular carcinoma. Sorafenib chemotherapy can be administered when nutritional status allows (16). There are still a large number of new targeted drugs in clinical trials, but the overall median overall survival (OS) of targeted therapy for advanced hepatocellular carcinoma is about 1 year. The objective response rate is low, and the associated adverse effects can involve multiple systems and organs throughout the body, which seriously affects the patient’s outcome. However, maxillofacial metastases are unique in that they can cause severe bleeding due to tumor pro-angiogenic factors and coagulation disorders, which can affect the survival rate and quality of life. Therefore, local excision and adjuvant radiation therapy can be considered for patients with isolated metastases, and the main aim is to reduce pain and masticatory function loss (17).

## 4 Conclusions

Condylar metastases from HCC are extremely rare and can easily be misdiagnosed as temporomandibular joint disorder (TMJD) or a primary tumor of the condyle without imaging and pathology diagnoses. Therefore, clinicians should pay due attention to the patient’s history and imaging, so as to reduce misdiagnosis. Patients with jaw metastasis from HCC have a poor prognosis and may be treated palliatively. The risk of surgery should be assessed by appropriate clinical examination before surgery. When the benefit far outweighs the risk, patients with severe pain and functional impairment may be treated with palliative surgery.

## Data availability statement

The original contributions presented in the study are included in the article/supplementary material. Further inquiries can be directed to the corresponding authors.

## Ethics statement

The studies involving human participants were reviewed and approved by The affiliated hospital of Qingdao University. The patients/participants provided their written informed consent to participate in this study. Written informed consent was obtained from the individual(s) for the publication of any potentially identifiable images or data included in this article.

## Author contributions

LC, YL, and JS were the patient’s surgeons. XL reviewed the literature and contributed to manuscript drafting. XL, NL and CF were responsible for the revision of the manuscript for important intellectual content. LC and CF contributed equally to this work and are co-corresponding authors. All authors contributed to the article and approved the submitted version.
